# A standardized catalogue of spectral indices to advance the use of remote sensing in Earth system research

**DOI:** 10.1038/s41597-023-02096-0

**Published:** 2023-04-08

**Authors:** David Montero, César Aybar, Miguel D. Mahecha, Francesco Martinuzzi, Maximilian Söchting, Sebastian Wieneke

**Affiliations:** 1 Remote Sensing Centre for Earth System Research (RSC4Earth), Leipzig University, Leipzig, 04103 Germany; 2 Image Processing Laboratory, University of Valencia, Valencia, 46980 Spain; 3 High Mountain Ecosystem Research Group, National University of San Marcos, 15081 Lima, Peru; 4 Helmholtz Centre for Environmental Research, Leipzig, 04318 Germany; 5 Center for Scalable Data Analytics and Artificial Intelligence (ScaDS.AI), Leipzig, 04105 Germany; 6 Image and Signal Processing Group, Leipzig University, Leipzig, 04109 Germany

**Keywords:** Environmental sciences, Biogeochemistry

## Abstract

Spectral Indices derived from multispectral remote sensing products are extensively used to monitor Earth system dynamics (e.g. vegetation dynamics, water bodies, fire regimes). The rapid increase of proposed spectral indices led to a high demand for catalogues of spectral indices and tools for their computation. However, most of these resources are either closed-source, outdated, unconnected to a catalogue or lacking a common Application Programming Interface (API). Here we present “Awesome Spectral Indices” (ASI), a standardized catalogue of spectral indices for Earth system research. ASI provides a comprehensive machine readable catalogue of spectral indices, which is linked to a Python library. ASI delivers a broad set of attributes for each spectral index, including names, formulas, and source references. The catalogue can be extended by the user community, ensuring that ASI remains current and enabling a wider range of scientific applications. Furthermore, the Python library enables the application of the catalogue to real-world data and thereby facilitates the efficient use of remote sensing resources in multiple Earth system domains.

## Background & Summary

The constant monitoring of surface processes is a prerequisite for understanding Earth System’s dynamics and functioning^1–6^. The big expansion of freely available remote sensing resources^6–8^ opens unprecedented opportunities for exploring interactions between the Earth’s surface and electromagnetic radiation. As new sensors come in ever higher spatial, temporal, and spectral resolutions, we constantly gain better insights into different Earth sub-systems. Surface materials have characteristic spectral signatures due to their physical properties and their interactions with electromagnetic radiation. As environmental effects can modify these interactions, spectral signatures give some insights into different surface processes, e.g., vegetation states^9^, urban expansion^5,10^, fires, and burned areas^11,12^. However, often one has to combine specific spectral regions of interest (i.e., spectral bands) in order to reduce unwanted (i.e., confounding effects) when studying a certain phenomenon or material^13–16^. Such combinations of bands, named spectral indices, supply condensed information on specific sub-systems, materials, or processes. For different application domains, multiple proposals for ideal spectral indices exist, and the field is constantly developing and testing novel spectral indices.

The most common application domain of spectral indices is the terrestrial biosphere, with a special focus on vegetation monitoring via Vegetation Indices (VI). Typically, VIs use spectral information (i.e., reflectance) from the visible and near-infrared (NIR) regions of the electromagnetic spectrum, exploiting the chlorophyll absorption bands and plant structural features for assessing vegetation states^17^. Along these lines, multiple VIs have been created, and the total number of indices has been growing steadily through time^17,18^. The most renowned and applied VI is the Normalized Difference Vegetation Index (NDVI)^13^, computed as the normalized difference between NIR and red reflectances, and generally used to assess vegetation greenness in space and time. Variants have also been developed by incorporating additional correction factors, such as the canopy background adjustment parameter in the Soil Adjusted Vegetation Index (SAVI)^14^, which reduces soil brightness effects. Some spectral indices have even been designed to serve as correction factors for scattering effects in Solar Induced Fluorescence (SIF), e.g., Fluorescence Correction Vegetation Index (FCVI)^19^ and Hyperspectral NIRv (NIRvH)^20^.

The goal of developing VIs specifically suited for a more accurate mapping of dynamic ecosystem processes, such as gross primary production (GPP, i.e., gross carbon uptake by plants)^6^, has led to a particularly wide variety of indices. The NIR Reflectance of Vegetation (NIRv)^21^ as well as the generalized Kernel NDVI (kNDVI)^22^ are examples of novel indices that aim to serve as a proxy for GPP. New sensors are capable of retrieving the reflectance in the red edge, which is highly sensitive to chlorophyll content due to the chlorophyll absorption shift to a longer wavelength. This led to a new set of indices around these bands. For instance, the Medium Resolution Imaging Spectrometer (MERIS) Terrestrial Chlorophyll Index (MTCI)^23^ was created from the red and red edge bands of the sensor on board the Envisat satellite and served as a proxy for estimating the Red Edge Position, also known as the Red Edge Point (REP), which is used for estimating canopy chlorophyll content. In particular, the Multispectral Instrument (MSI) on board Sentinel-2 satellites enables the computation of red edge indices, such as the Inverted Red Edge Chlorophyll Index (IRECI) as well as the Sentinel-2 REP (S2REP)^24^.

Indices for other application domains also contribute to the growing list of spectral indices. Water Indices (WI) generally use reflectance from the green, NIR and Shortwave Infrared (SWIR) bands to discriminate water bodies from other land cover types such as soil, vegetation, or constructed surfaces^25^. One of the most used WIs is the Normalized Difference Water Index (NDWI)^16^, computed as the normalized difference between green and NIR reflectances for delineating water bodies. Variants of this index, such as the Modified NDWI (MNDWI)^26^, have been created by replacing the NIR for SWIR reflectance, particularly reducing the influence of the noise produced by built-up areas. The number of WIs is also increasing due to the launching of new satellites and enhanced sensors. The Multi-Band Water Index (MBWI)^27^ was developed for images of the Operational Land Imager (OLI) sensor of Landsat-8, while the Sentinel-2 Water Index (S2WI)^28^ was created by taking advantage of the red edge bands in Sentinel-2 MSI imagery.

Modifications of existent indices as well as the development of new sensors with additional bands or different spectral characteristics are the main reasons for the growth of new spectral indices in other application domains. Besides WIs, indices for identifying burned areas and active fires are being expanded. For instance, the Burned Area Index (BAI)^29^, one of the most used indices for detecting fire-affected areas, initially developed for the Advanced Very-High-Resolution Radiometer (AVHRR) instrument, has recently been modified to match Sentinel-2 MSI spectral bands (BAIS2)^30^. BAIS2 uses red edge bands as well as the narrow NIR band. Another example is snow and ice cover indices. The Normalized Difference Snow Index (NDSI)^15^ was developed for the Landsat’s Thematic Mapper (TM) and the Moderate Resolution Imaging Spectroradiometer (MODIS) instruments using the green and SWIR bands, while one of the newest indices, the Snow Water Index (SWI)^31^, developed for Landsat-8 OLI and Sentinel-2 MSI instruments, additionally use the NIR band aiming to discriminate better between snow cover and water bodies. This is also the case of Urban and Soil Indices. The Urban Index (UI)^32^, developed to map urban areas and investigate urban density and socio-economic variables, was constructed using images from the Landsat TM instrument. Contemporaneous indices such as the Dry Built-Up index (DBI)^33^ use the Thermal Infrared (TIR) band from the Landsat OLI instrument to improve the discrimination of built-up areas and bare soil in dry climates. Likewise, Soil Indices like the Bareness Index (BaI)^34^ were created for detecting the degree of bareness on the surface using bands from Landsat TM. By using bands from Landsat OLI, new indices such as the Modified Bare Soil Index (MBI)^35^ and the Enhanced MBI (EMBI)^5^ have been developed, which particularly suppress built-up areas when detecting bare soil areas.

The steady growth of spectral indices across application domains inevitably calls for a central repository, gallery, or catalogue, where the remote sensing community can explore the vast amount of indices for specific applications or analyses. Arguably, one of the most comprehensive catalogues of spectral indices is the Index DataBase (IDB)^36,37^, which has condensed several indices in a relational data model, specially VIs, as well as multiple attributes such as their formula, acronym, original name, used bands for different sensors and a collection of articles that cite most of the indices. The IDB includes a JavaScript Object Notation (JSON) Application Programming Interface (API) for querying spectral indices, which can be used upon request. Specifically for VIs, the review article by Xue *et al*., (2017)^18^ presents a comprehensive review of several VIs, including their formulas and references. Proprietary software, such as ArcGIS and ENVI, count with their own spectral indices gallery (see https://pro.arcgis.com/en/pro-app/latest/help/data/imagery/indices-gallery.htm and https://www.l3harrisgeospatial.com/docs/alphabeticallistspectralindices.html, respectively). While they exhibit formulas, descriptions, and references, and can be accessed freely online, they require a license to compute them. There are few open-source projects providing tools to calculate spectral indices. Examples include the RadiometricIndices application from Orfeo Toolbox^38^ (see https://www.orfeo-toolbox.org/CookBook/Applications/app_RadiometricIndices.html); the Vegetation Indices tools from SAGA-GIS^39^ (see https://saga-gis.sourceforge.io/saga_tool_doc/8.4.1/imagery_tools.html); the spectralIndices function from RStoolbox (see https://bleutner.github.io/RStoolbox/rstbx-docu/spectralIndices.html), which is written in C and works with raster objects in R; the nightmares package, which also leverages the computation of spectral indices inside R (see https://cran.r-project.org/web/packages/nightmares/index.html); and the multispectral module from xarray-spatial (see https://xarray-spatial.org/reference/multispectral.html), which works with dask for xarray.DataArray objects^40^ in Python. However, all the above mentioned open-source projects implement outdated sets of hard-coded indices that are not connected to a standardized open community catalogue.

Despite the fact that numerous resources have been developed for querying and computing spectral indices, multiple barriers still abridge the remote sensing community from exploiting them: The first obstacle is the outdated proprietary-dependent set of indices. The lack of novel spectral indices prevents users from making the most of spectral features that are offered by today’s instruments. This hurdle grows bigger as multiple resources have a limited amount of indices in their catalogues, depriving users not just from novel, but also from traditional, as well as rare spectral indices. As the number of indices continues to increase, the remote sensing community requires an easy way to implement indices on their own on demand. The second obstacle is that spectral indices toolboxes are not connected to an existent open-source catalogue and indices are usually hard-coded inside specific modules. While this is not a major issue for open-source resources where the community can contribute by adding new indices, it is a stumbling block for non-developers who need a quick way to compute spectral indices that are not included yet. Vice versa, catalogues that are not linked to a toolbox are not entirely harnessing their potential by providing an application that the community may use to query and compute the included spectral indices.

The aim of this paper is to present “Awesome Spectral Indices” (ASI), an open community catalogue for handling spectral indices in Earth System research that addresses the research gap described above. ASI comprises a spectral indices catalogue, which is connected to a Python library. The catalogue of spectral indices is a free-to-access online open-source repository that follows a basic key-value structure and a simple standard for spectral indices in different application domains. The catalogue’s straightforward architecture allows the community to request or add new indices as they become available. The Python library, named spyndex, uses the catalogue to create an user-friendly module for querying and automatically computing spectral indices for multiple Python objects (e.g. arrays and data frames). ASI leverages the data model structure as well as a Python library for easy management of spectral indices for their use in the analysis of Earth’s surface processes using remote sensing data streams.

## Methods

### Catalogue design

#### Data model

The catalogue is designed as a simple nested key-value model (Fig. 2b) that stores attributes for multiple spectral indices. For simplicity, the set of spectral indices (represented by the acronyms as string objects) is defined as $\Psi =\{{\psi }_{1},...,{\psi }_{n}\}$, where *ψ* is a spectral index and *n* is the number of indices in the list. The set of application domains is defined as *O* = {≪vegetation≫, ≪water≫, ≪burn≫, ≪snow≫, ≪urban≫, ≪soil≫, ≪radar≫, ≪kernel≫}. The catalogue is formally defined as $C=\{({\psi }_{1},{A}_{{\psi }_{1}}),...,({\psi }_{n},{A}_{{\psi }_{n}})\}$, where *C* is the catalogue, *ψ* is a spectral index and $\psi \in \Psi$, *A*_*ψ*_ is the set of attributes of *ψ* and *n* is the number of indices in *C*. Each index *ψ* is the primary key of the model and the corresponding value *A*_*ψ*_ is a set of 9 attributes represented by a secondary key-value model (Fig. 2c) such that

1 $${A}_{\psi }=\left\{\begin{array}{l}(\ll {\rm{short}}\_{\rm{name}}\gg ,\psi )\\ (\ll {\rm{long}}\_{\rm{name}}\gg ,{\eta }_{\psi })\\ (\ll {\rm{application}}\_{\rm{domain}}\gg ,{o}_{\psi })\\ (\ll {\rm{formula}}\gg ,{E}_{\psi })\\ (\ll {\rm{bands}}\gg ,{V}_{\psi })\\ (\ll {\rm{platforms}}\gg ,{Z}_{\psi })\\ (\ll {\rm{reference}}\gg ,{r}_{\psi })\\ (\ll {\rm{date}}\_{\rm{of}}\_{\rm{addition}}\gg \,,{d}_{\psi })\\ (\ll {\rm{contributor}}\gg \,,{c}_{\psi })\end{array}\right\}$$

where *ψ* is the short name (i.e., acronym) of the index and $\psi \in \Psi$, ${\eta }_{\psi }$ is the long name (i.e., original name) of the index, ${o}_{\psi }$ is the application domain of the index and ${o}_{\psi }\in O$, ${E}_{\psi }$ is the expression of the index, ${V}_{\psi }$ is the set of operands in ${E}_{\psi }$ and ${V}_{\psi }\subseteq V$ (see Expressions section), ${Z}_{\psi }$ is the set of platforms with the required bands for the index computation, ${r}_{\psi }$ is the URL or DOI of the reference of the index, ${d}_{\psi }$ is the date of addition to *C*, and ${c}_{\psi }$ is the URL of the contributor’s GitHub profile.

Contributors must fill all attributes of an index except ${V}_{\psi }$ and ${Z}_{\psi }$, which are extracted automatically after ${E}_{\psi }$ is validated. The set of platforms ${Z}_{\psi }$ is filled up taking ${V}_{\psi }$ into account. If a platform $z\in Z$ counts with the required bands for a spectral index computation, that is, ${V}_{\psi }\subseteq {V}_{z}$, the platform *z* is added to ${Z}_{\psi }$. As of version 0.4.0, the set of platforms *Z* is {≪MODIS≫, ≪Landsat-TM≫, ≪Landsat-ETM+≫, ≪Landsat-OLI≫, ≪Sentinel-2≫, ≪Sentinel-1 (Dual HH-HV)≫, ≪Sentinel-1 (Dual VV-VH)≫, ≪Planet-Fusion≫}. Note that platforms with the same sensors (or spectral bands) were grouped (e.g. ≪Landsat-TM≫ refers to the group of the Landsat 4 and 5 satellites).

#### Expressions

An expression *E* is a string object that can be parsed as a representation of an operation (e.g. ≪a + b≫ is an expression representing the *a* + *b* mathematical operation, where ≪a≫ and ≪b≫ are operands, and ≪+≫ is an operator). Given this, a well defined valid expression can be easily evaluated in any programming language, leveraging the need to re-write each spectral index for all languages where spectral indices can be implemented.

To establish a standard expression evaluation model, all possible expression operands *V* and operators *G* in spectral indices must follow a standard naming. The complete set of standards is given by $S=V\cup G$. Given this, bands, additional parameters, band dependent parameters, and common operators require a well defined standard. *G* was created from the standard operators ≪+≫, ≪−≫, ≪*≫, and ≪/≫, that were used for addition, subtraction, multiplication and division operations, respectively. The ≪**≫ operator was used for exponentiation operations instead of ≪ˆ≫ to make it compatible with Python. *V* was created from the bands of popular multispectral sensors in satellite remote sensing platforms, therefore, each standard name corresponds to a band in one of these platforms (Table 2). The standard names were kept as short as possible, representing them in most of cases as a single character in capital letters to avoid large string expressions. When more than one band for the same region is encountered, a consecutive number is added at the end of the standard name. For Radar indices, the standards ≪HH≫, ≪HV≫, ≪VV≫, and ≪VH≫ were created for the corresponding polarization mode no matter the wavelength (e.g. C-band of the C-SAR instrument on Sentinel-1 satellites).

This standardization accounts for spectral indices that do not require any additional parameters, such as the NDVI:

2 $${\rm{NDVI}}=\left({\rho }_{{\rm{NIR}}}-{\rho }_{{\rm{red}}}\right)/\left({\rho }_{{\rm{NIR}}}+{\rho }_{{\rm{red}}}\right)$$

where *ρ*_*i*_ is the reflectance in band *i*. Equation 2 is represented by ${E}_{{\rm{NDVI}}}=\ll ({\rm{N}}-{\rm{R}})/({\rm{N}}+{\rm{R}})\,\gg$ using *S*, where *V*_NDVI_ = {≪N≫, ≪R≫,}. Nevertheless, additional parameters must also be standardized for spectral indices that require them. These parameters are standardized according to the notation used in the original index, keeping the standard names as similar as possible to the original parameters no matter the length, and using the name of greek letters when encountered, e.g., the weighting coefficient *α* in the Wide Dynamic Range Vegetation Index (WDRVI)^41^ is represented by the standard name ≪alpha≫ (Table 3). Band dependent parameters, such as band wavelengths *λ*_*i*_ and kernels *K*(*a, b*) were standardized by using the common names ≪lambdaA≫ and ≪kAB≫, where ≪lambdaA≫ is the wavelength of band A and ≪kAB≫ is a kernel function of A and B (being A and B band reflectances or other additional parameters).

By using these additional standard names, spectral indices like the Hyperspectral Near-Infrared Reflectance of Vegetation (NIRvH2)^20^ are also accounted for. Given this, the equation:

3 $${\rm{NIRvH}}2={\rho }_{{\rm{NIR}}}-{\rho }_{{\rm{red}}}-k\times \left({\lambda }_{{\rm{NIR}}}-{\lambda }_{{\rm{red}}}\right)$$

where *k* is the soil slope between red and NIR and *λ*_*i*_ is the wavelength of band *i,* is represented by the expression ${E}_{{\rm{NIRvH2}}}=\ll {\rm{N}}-{\rm{R}}-{{\rm{k}}}^{\ast }({\rm{lambdaN}}-{\rm{lambdaR}})\gg$, where *V*_NIRvH2_ = {≪N≫, ≪R≫, ≪k≫, ≪lambdaN≫, ≪lambdaR≫}. The ≪N≫ and ≪R≫ characters in the ≪lambdaN≫ and ≪lambdaR≫ expressions have no conflicts with the *ρ*_NIR_ and *ρ*_red_ standards since the complete expression is parsed as a single variable as long as it doesn’t have any spaces or operators. This principle also applies to generalized kernel indices such as the kNDVI:

4 $${\rm{kNDVI}}=\frac{K({\rho }_{{\rm{NIR}}},{\rho }_{{\rm{NIR}}})-K({\rho }_{{\rm{NIR}}},{\rho }_{{\rm{red}}})}{K({\rho }_{{\rm{NIR}}},{\rho }_{{\rm{NIR}}})+K({\rho }_{{\rm{NIR}}},{\rho }_{{\rm{red}}})}$$

where *K*(*ρ*_*i*_, *ρ*_*j*_) is a kernel function of the reflectances in bands *i* and *j*. Equation 4 is represented by the expression ${E}_{{\rm{kNDVI}}}=\ll \,({\rm{kNN}}-{\rm{kNR}})/({\rm{kNN}}+{\rm{kNR}})\gg$ using *S*, where *V*_kNDVI_ = {≪kNN≫, ≪kNR≫}. This standard has no conflicts with the standard of raw reflectances and allows the optimization of generalized kernel indices by enabling the free choice of the kernel function as well as their parameters.

#### Release

After validation (see Technical Validation), all indices are exported as a JSON file by keeping the original nested key-value model and the GitHub repository is updated. Additionally, the JSON file is used to convert the nested key-value model to a relational model exported as a CSV file. This model permits the use of the catalogue as a tabular structure that can be easily read as a data frame datatype in many programming languages (e.g. R and Julia). Furthermore, it can be loaded as a table using a Relational Database Management System (RDBMS) such as PostgreSQL, allowing the use of the domain specific SQL language for querying the catalogue, and even as a spreadsheet in proprietary software such as Microsoft Excel. After being updated, a new version of the GitHub repository is officially released. All sources are matched to their relevant indices in the main data files using the ≪reference≫ attribute, and cited here^5,9–16,19–35,41–166^.

### Python library design

spyndex is an open-source Python package hosted in GitHub that works as the Python library for ASI (Fig. 2f). This package allows users to query and compute spectral indices from the catalogue. The spectral indices are automatically updated in the spyndex repository once they are released by keeping a local copy of the JSON file provided by the catalogue itself (Fig. 2i). This allows users to access and compute spectral indices even if they don’t have an internet connection. Nevertheless, users can still use the most recent version of the catalogue for spectral indices computation if an internet connection is provided.

#### Querying spectral indices and parameters

The local copy of the JSON file is used to create a new class named SpectralIndex. This class stores the attributes of each index in the catalogue and saves them as its own attributes. Additionally, this class counts with a method for computing the index by passing the required parameters as a dictionary object or as keywords arguments. By using this class, each index in the local copy of the catalogue is transformed to a SpectralIndex object that can be accessed and computed directly. All transformed indices are then stored in a SpectralIndices object, which is constructed by inheritance from the Box class^167^, allowing indices querying by using dot notation and text auto-completion (Fig. 2h).

Standards for band reflectances also have a new specific class for querying information relative to different satellite platforms and their specifications. The Band class is created to store the ≪standard≫ and ≪long_name≫ attributes, which users can access from the specific band. Additionally, the attributes ≪sentinel2a≫, ≪sentinel2b≫, ≪landsat4≫, ≪landsat5≫, ≪landsat7≫, ≪landsat8≫, ≪landsat9≫ and ≪modis≫ are also created for each band, containing information on the band name, band number, center wavelength (nm) and bandwidth (nm) for each specific platform in a class named PlatformBand. The Band class is useful for the computation of indices that require the specific platform band wavelengths *λ*_*i*_ such as the NIRvH2 and the Normalized Difference Greenness Index (NDGI)^159^. All bands are then stored in a Bands object, also inherited from the Box class to allow the use of dot notation and auto-completion (Fig. 2h).

Additional parameters (Table 3) that also follow a standard naming are included in a new class named Constant. This class stores the ≪standard≫, ≪long_name≫ and ≪default≫ attributes, representing the standard name, the original name and the default value of the parameter. Furthermore, specific kernel parameters were also included here for the calculation of kernel indices. These kernel parameters include the length-scale parameter *σ* for the Radial Basis Function (RBF) Kernel *K*_RBF_ (Eq. 7), and the polynomial degree *p* and the trade-off parameter *c* in the Polynomial Kernel *K*_poly_ (Eq. 6). By using this class, users can decide whether to use the default values for the additional parameters, or to optimize them by using any other value or a compatible Python object. This becomes handy when computing indices that require additional parameters such as the Enhanced Vegetation Index (EVI)^90^ and the Optimized Chlorophyll Vegetation Index (OCVI)^141^, among others. All parameters are stored in a Constants object inherited from a Box class for their access using dot notation and text auto-completion (Fig. 2h).

#### Computing spectral indices

In Python, a single string expression can be evaluated as *f*(*E*, *P*), where *f* is the evaluation function, *E* is the expression to evaluate, and *P* is a dictionary of local variables that serve as inputs for undefined variables in *E*. Since ${E}_{\psi }\in {A}_{\psi }$ is a valid expression that was curated in the catalogue validation process, it can be used without any modification to compute a spectral index by being evaluated. This evaluation is performed by passing the required parameters ${V}_{\psi }$ for the formula computation as interoperable Python objects that support mathematical overloaded operators. This means that the evaluation process is successful for objects with mathematical overloaded operators that perform the actual mathematical operations (e.g. a ≪+≫ operator performs an addition for two integers), but it will fail for objects with mathematical overloaded operators that do not perform the actual mathematical operations (e.g. a ≪+≫ operator performs a concatenation for two lists).

By this definition, spectral indices can be computed by taking advantage of the expression evaluation process in Python. A single spectral index *ψ* can then be evaluated by $f({E}_{\psi },{P}_{\psi })$, where ${E}_{\psi }$ is the expression of the spectral index *ψ* and ${P}_{\psi }$ is the dictionary of parameters required to evaluate the index expression such that ${P}_{\psi }=\left\{({\upsilon }_{1},{\rho }_{1}),...,({\upsilon }_{n},{\rho }_{n})\right\}$, where ${\upsilon }_{i}\in {V}_{\psi }$ and *ρ*_*i*_ is the reflectance of band *i* as a compatible Python object. For example, to calculate the NDVI (Eq. 2) it is required to compute $f({E}_{{\rm{NDVI}}},{P}_{{\rm{NDVI}}})$, where ${E}_{{\rm{NDVI}}}=\ll ({\rm{N}}-{\rm{R}})/({\rm{N}}+{\rm{R}})\gg$, and ${P}_{{\rm{NDVI}}}=\{(\ll {\rm{R}}\gg \,,{\rho }_{{\rm{red}}}),(\ll {\rm{N}}\,\gg \,,{\rho }_{{\rm{NIR}}})\}$. Here, *P*_NDVI_ is a dictionary where the standard names ≪N≫ and ≪R≫ are keys and their values *ρ*_*i*_ can be either single values or *n*-dimensional arrays.

This computation process is leveraged by the function computeIndex, which takes the acronym of the index *ψ* and the dictionary of parameters ${P}_{\psi }$ for the index computation as inputs. By doing this, computeIndex works on a higher level by computing $g(\psi ,{P}_{\psi })$ instead of $f({E}_{\psi },{P}_{\psi })$, which leverages the need of writing the complete expression by just using the index acronym. ${E}_{\psi }$ is taken from the ≪formula≫ attribute in the local copy of the catalogue, although it can be requested directly from the online version in the updated catalogue. ${P}_{\psi }$ is passed by the user and must contain the same keys as ${V}_{\psi }$. If this is not complied, the function will raise an error. In addition, the dictionary of parameters is interchangeable with keyword arguments for a more fluid scripting.

Multiple indices can be computed at once by passing a list of index acronyms ${\Psi }_{s}\subseteq \Psi$ to the function. In this case, the dictionary of parameters is now ${P}_{{\Psi }_{s}}={\cup }_{i=1}^{n}{P}_{{\psi }_{i}}$, where ${\psi }_{i}\in {\Psi }_{s}$, and *n* is the number of indices in ${\Psi }_{s}$. The function can then be computed as $g\left({\Psi }_{s},{P}_{{\Psi }_{s}}\right)$, which is easier and more efficient than computing $g\left({\psi }_{i},{P}_{{\psi }_{i}}\right)$ for all ${\psi }_{i}\in {\Psi }_{s}$. For example, in order to compute the Chlorophyll Index Red Edge (CIRE)^78^, where ${P}_{{\rm{CIRE}}}=\{(\ll {\rm{RE}}1\,\gg \,,{\rho }_{{\rm{RE}}1}),(\ll {\rm{N}}\,\gg \,,{\rho }_{{\rm{NIR}}})\}$, and the NDVI, where ${P}_{{\rm{NDVI}}}=\{(\ll {\rm{R}}\,\gg \,,{\rho }_{{\rm{red}}}),(\ll {\rm{N}}\,\gg \,,{\rho }_{{\rm{NIR}}})\}$, the required dictionary of parameters is now passed as ${P}_{{\rm{C}}{\rm{I}}{\rm{R}}{\rm{E}}}\cup {P}_{{\rm{N}}{\rm{D}}{\rm{V}}{\rm{I}}}=\{(\ll {\rm{R}}\gg ,{\rho }_{{\rm{r}}{\rm{e}}{\rm{d}}}),(\ll {\rm{R}}{\rm{E}}1\gg ,{\rho }_{{\rm{R}}{\rm{E}}1}),(\ll {\rm{N}}\gg ,{\rho }_{{\rm{N}}{\rm{I}}{\rm{R}}})\}$, and both indices can be computed at once by passing ${\Psi }_{{\rm{s}}}=\{\ll {\rm{CIRE}}\,\gg \,,\ll {\rm{NDVI}}\gg \}$.

Generalized kernel indices can also be computed using computeIndex. However, to allow the optimization of kernel parameters, the computeKernel was created. This function permits users to compute a kernel *K*(*a, b*), where *K* is a kernel function, and α and *b* can be whether reflectances or additional parameters. As an illustrative example, the kNDVI computation requires ${P}_{{\rm{kNDVI}}}=\{(\ll {\rm{kNN}}\,\gg \,,K({\rho }_{{\rm{NIR}}},{\rho }_{{\rm{NIR}}})),(\ll {\rm{kNR}}\,\gg \,,K({\rho }_{{\rm{NIR}}},{\rho }_{{\rm{red}}}))\}$, where *K* can be any kernel function computed using the computeKernel method. Three kernel functions are implemented in spyndex. These kernels are (1) the Linear Kernel:

5 $${K}_{{\rm{linear}}}(a,b)=ab$$

(2) the Polynomial Kernel:

6 $${K}_{{\rm{poly}}}(a,b)={(ab+c)}^{p}$$

where *c* is a trade-off parameter between higher and lower order terms in the polynomial and *p* is the polynomial degree, and (3) the RBF Kernel:

7 $${K}_{{\rm{RBF}}}(a,b)=\exp \left(-\frac{{(a-b)}^{2}}{2{\sigma }^{2}}\right)$$

where *σ* is the length-scale parameter. The *K*_RBF_ is suggested for the kNDVI computation with $\sigma =0.5({\rho }_{{\rm{NIR}}}+{\rho }_{{\rm{red}}})$^22^. However, the *σ* parameter can be chosen by the user in computeKernel, allowing the optimization of *K*_RBF_. This principle also applies for the *c* and *p* parameters in *K*_*poly*_.

Likewise, this is useful for the creation and optimization of generalized kernel indices. Since all indices can be kernelized^22^, the computeKernel function can be used for the required bands in kernelized indices that are being created and are not part of the catalogue yet. As an illustrative example, the Green Normalized Difference Vegetation Index (GNDVI)^74^ can be kernelized as:

8 $${\rm{kGNDVI}}=\frac{K({\rho }_{{\rm{NIR}}},{\rho }_{{\rm{NIR}}})-K({\rho }_{{\rm{NIR}}},{\rho }_{{\rm{green}}})}{K({\rho }_{{\rm{NIR}}},{\rho }_{{\rm{NIR}}})+K({\rho }_{{\rm{NIR}}},{\rho }_{{\rm{green}}})}$$

and all kernels in this equation can be easily computed by passing the corresponding *ρ*_NIR_ and *ρ*_green_ reflectances to computeKernel and selecting the desired kernel function with the required parameters for optimization.

## Data Records

**Fig. 1:**
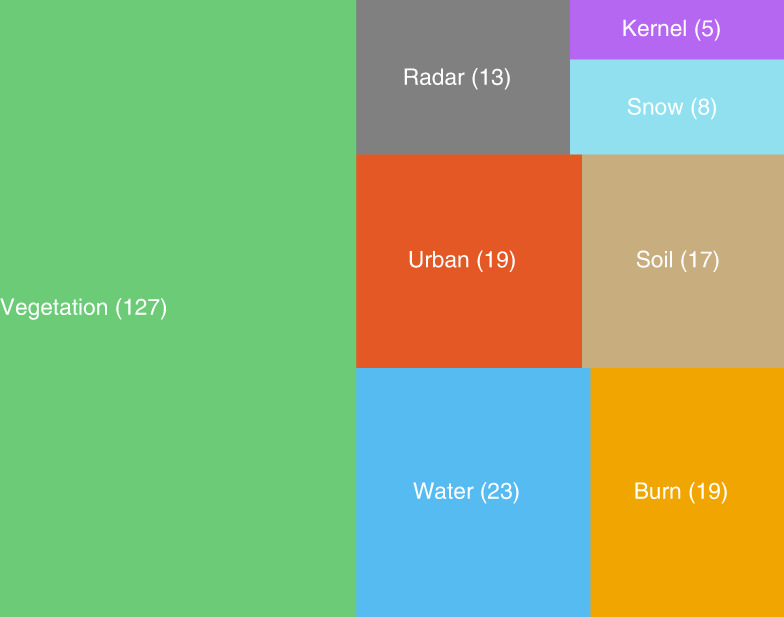
Treemap of the count of spectral indices per application domain (as of version 0.4.0). The number of indices is reported inside parentheses.

**Fig. 2:**
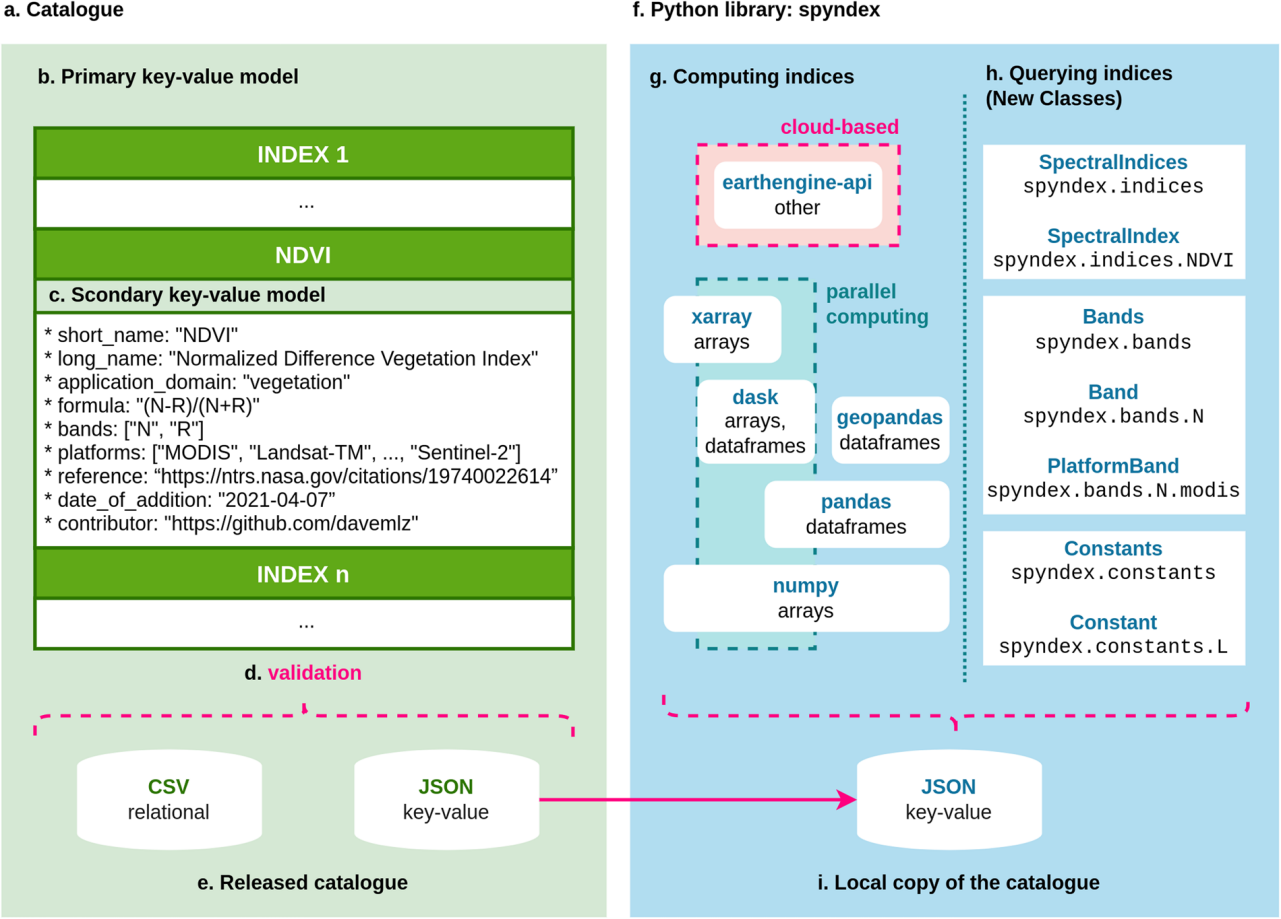
A simplified diagram of the structure and workflow supported by ASI. (**a**) Simplified representation of the nested key-value data model, (**b**) primary key-value model representing indices and the set of attributes, (**c**) secondary key-value model representing the values for each attribute, (**d**) validation process of the catalogue before releasing, (**e**) final formats of the released catalogue, (**f**) simplified representation of the Python library, (**g**) libraries and data types suitable for spectral indices computation, (**h**) new classes with usage examples for querying spectral indices as well as their attributes and additional parameters, and (**i**) local copy of the JSON file used as base for the library.

The ASI Catalogue (Fig. 2a) is a curated growing collection of currently 231 spectral indices (as of version 0.4.0) widely used in remote sensing. The catalogue is divided into 8 groups, which mostly represent specific application domains (Fig. 1), namely: vegetation, water, burn, snow, urban, radar, soil, and kernel indices. Note that radar and kernel indices reflect specific methodological approaches. For instance, indices from any Earth system application domain could be converted to generalized kernel indices^22^. However, we decided to create a group in its own right for indices of this kind since their mathematical structure involves a free parameterization.

The complete set of spectral indices per application domain (as of version 0.4.0) in JSON and CSV formats is available online via Zenodo^168^. The JSON file stores the catalogue using a key-value model, with the acronym of each index as the key and the set of 9 attributes as the value. The CSV file stores the catalogue as a relational model, with each row representing an index and the 9 attributes as columns. The data types for each attribute are listed in Table 4. Note that by modifying the original key-value model in the CSV file, the ≪bands≫ and ≪platforms≫ attributes lose their array structure and additional string management must be done to recover it while keeping the relational model.

In addition to the set of spectral indices, two JSON files containing multiple attributes for the standard of each band as well as the additional parameters are included in the Zenodo repository. The JSON file for the bands includes the ≪long_name≫, ≪short_name≫, ≪platforms≫, ≪min_wavelength≫, and ≪max_wavelength≫ attributes. These attributes describe each one of the bands, including their spectral range and the set of platforms where the band is found. Furthermore, it includes the ≪common_name≫ attribute, which creates a direct mapping of the ASI standard to the Electro-Optical Extension Specification (see https://github.com/stac-extensions/eo/) for SpatioTemporal Asset Catalogues (STAC). The JSON file for the additional parameters includes the ≪default≫, ≪description≫, and ≪short_name≫ attributes. These attributes describe each one of the additional parameters, including the default value of the parameter in the original paper where it is found.

## Technical Validation

### Catalogue validation

The living catalogue is continuously updated by the main developers based on rigorous literature reviews. Users can request the addition of new indices by using issues or pull requests. All spectral indices are validated using pydantic^169^ (Fig. 2d), a Python package for data validation using type annotations. For this validation step, all indices must comply with the following constraints before being added to the catalogue: (1) All attributes must be filled with a string datatype, (2) *ψ* must be written in capital letters if possible and must not contain spaces, (3) *E*_*ψ*_ must be a valid expression and must use items from the set of standards *S* (see Expressions section), (4) *d*_*ψ*_ must be in “YYYY-MM-DD” format, (5) *c*_*ψ*_ must be a valid GitHub profile URL or an email, and (6) ${o}_{\psi }\in O$. If the input indices do not comply with the above mentioned constraints the system raises an error and the catalogue is not updated until corrected. In the case of source references, the main developers review the reference provided by the contributor of an index. If the reference is not the original source of the index (e.g., a published paper, a poster, or grey literature), the index is not added to the catalogue until the original source is provided.

### Compatibility with python objects

The expression evaluation taking mathematical overloaded operators into account makes it possible for different types of Python objects to be compatible with spyndex (Table 1). First, the primitive numerical Python built-in types int (integers) and float (floating points) are compatible with mathematical overloaded operators and thus compatible with spyndex. This primitive compatibility allows most Python objects that are built on top of them to be compatible with spyndex. Second, the primitive data types in the numpy package^170^ (e.g., numpy.int and numpy.float) are also compatible with mathematical overloaded operators and therefore most Python objects built on top of numpy are also compatible with spyndex (Fig. 2g).

**Table 1:** Supported Python objects.

Module	Class	Type	Computing
Python Built-in	int	Primitive	Serial
	float	Primitive	Serial
numpy	int	Primitive	Serial
	float	Primitive	Serial
	ndarray	Array	Serial
xarray	Dataset	Array	Serial
	DataArray	Array	Serial
pandas	Series	Array	Serial
	DataFrame	Tabular	Serial
geopandas	Series	Array	Serial
	GeoDataFrame	Tabular	Serial
earthengine-api	Image	Other	Parallel
	Number	Other	Parallel
dask	Array	Array	Parallel
	DataFrame	Tabular	Parallel

This compatibility with the primitives of Python and numpy makes spyndex compatible with the most used objects: arrays and data frames. The primitive array objects can be created through numpy and they can be *n*-dimensional. These primitive arrays are the source of data arrays in the xarray package^40^, which allows the creation and manipulation of labelled data arrays and datasets. This is extremely useful for images with 3 dimensions {columns, rows, bands}, or data cubes with 4 dimensions {columns, rows, bands, time}. By using xarray.DataArray objects as inputs in a spectral index calculation, the result will be another xarray.DataArray with the same dimensions as the input arrays. If more than one index is calculated at the same time, by default, the result is a xarray.DataArray with an extra dimension named ≪index≫ and the acronyms of the indices as labels.

Additionally, spyndex is also compatible with data frames in the pandas package^171^, which allows the creation and manipulation of tabular data, and geopandas^172^, which adds a spatial dimension to tabular data in pandas. Usually, the input data consist of pandas.Series or geopandas.Series, and the result would be another series with the same length when one index is computed. When multiple indices are computed, by default, the result is a pandas.DataFrame where each column corresponds to a spectral index.

This compatibility enables the direct use of SpatioTemporal Asset Catalogues (STAC) for spectral indices computation with spyndex, allowing the querying of satellite imagery from a STAC provider (e.g., Planetary Computer) and the direct computation of spectral indices for the requested images according to the data type in which the data is stored. Additionally, spyndex also extends the compatibility to Google Earth Engine (GEE) objects for its Python API^8^. GEE is a cloud-based geoprocessing service that counts with a vast catalogue of satellite imagery that users can process online. Originally, GEE objects, such as ee.Image and ee.Number, are not compatible with mathematical overloaded operators. However, they are extended through the eemont package^173^. This permits the spyndex compatibility with GEE objects for large spectral indices computations in the cloud.

Other objects can also be compatible with spyndex if mathematical overloaded operators are implemented for the object’s class. This is useful for users that aim to create their own classes or modules. By emulating numeric types, the created classes can serve as inputs for objects that are compatible for spectral indices computation. If the new classes are created by inheritance of classes that already have overloaded operators, the new classes are automatically compatible with spyndex (e.g., a new class named Signature that inherits from numpy.ndarray and that represents a spectral signature can be used to compute spectral indices with spyndex).

## Usage Notes

The potential of the ASI catalogue can be exploited to boost computations of spectral indices from real-world data using parallel computing and multidimensional data management through the spyndex Python library. Nevertheless, the machine readable catalogue can be used in further programming languages with a special potential for Earth system research. Furthermore, advanced applications involving hyperspectral indices or Machine Learning (ML) pipelines can also be exploited with ASI.

### Python library usage examples

**Fig. 3:**
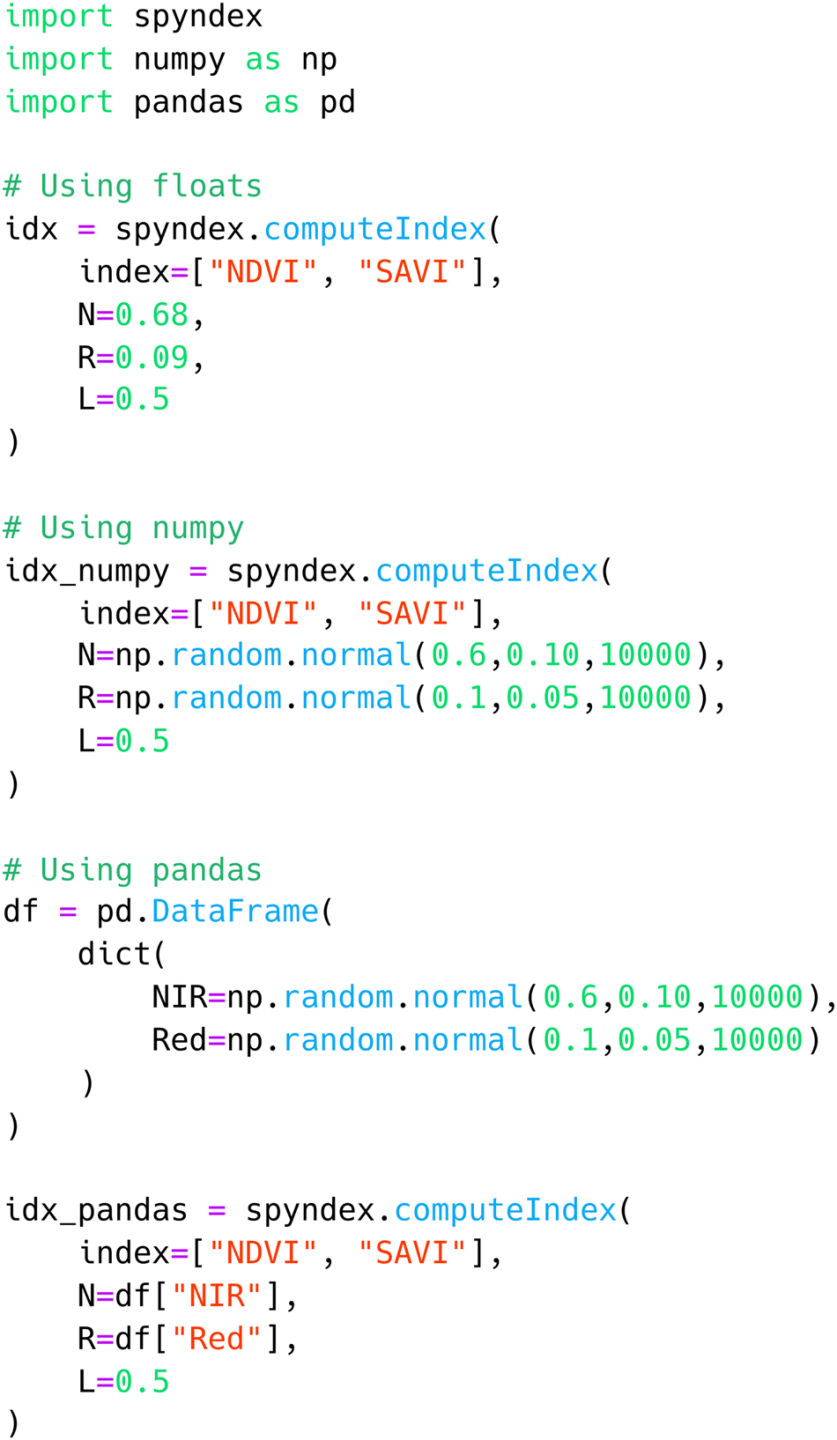
Example code for computing NDVI and SAVI for float, numpy.ndarray, and pandas.Series objects. Both indices can be computed at the same time with the same input values. Furthermore, additional parameters such as the Canopy Background Adjustment L can be passed as a single constant value. Note that spyndex returns an object of the same type as the input objects.

**Fig. 4:**
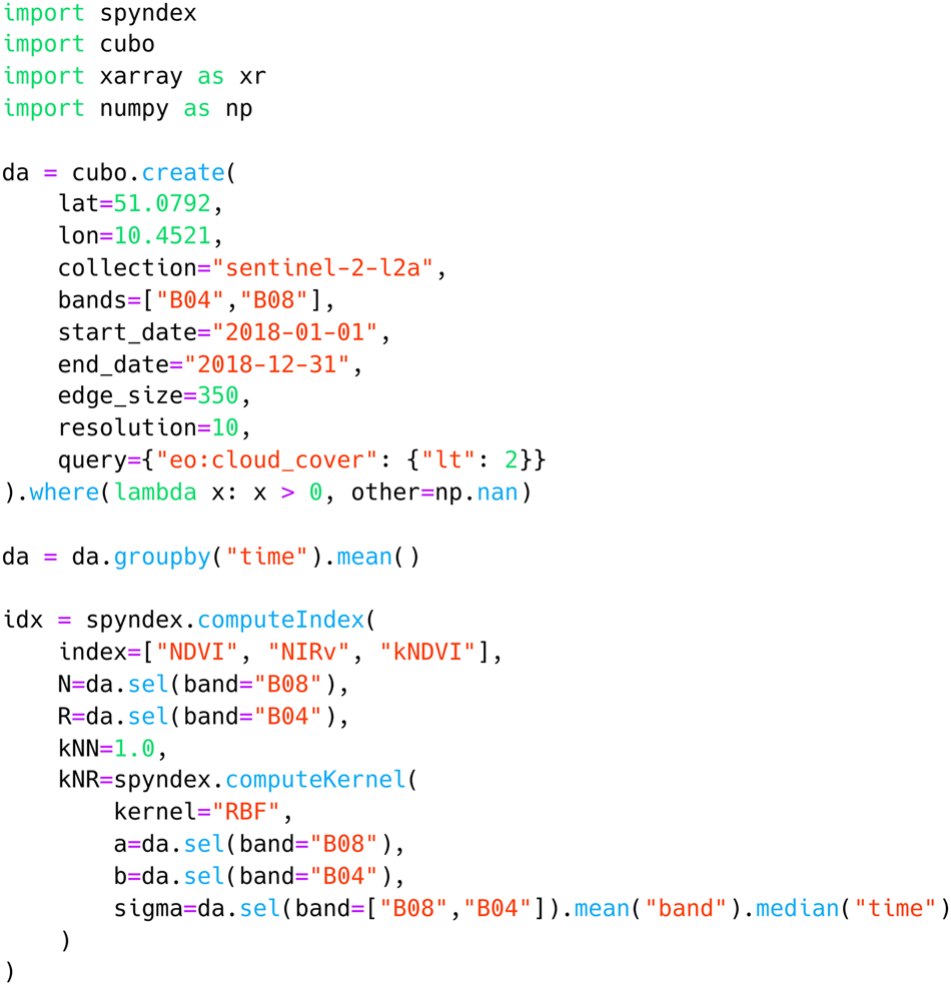
Example code for computing NDVI, NIRv, and kNDVI for a xarray.DataArray object. First, a Sentinel-2 L2A data cube is retrieved as a data cube via the cubo Python package. This data cube is a chunked xarray.DataArray with 4 dimensions: ≪bands≫, ≪time≫, ≪x≫, and ≪y≫. The ≪bands≫ dimension contains the labeled data for NIR and red surface reflectance values, therefore, it is used to select the values for each operand in the computation. Furthermore, this example displays how to compute a kernel for kernel indices such as the kNDVI. Note that the ≪kNN≫ operand was set to 1.0 since *K*_RBF_ was the selected kernel and *K*_RBF_(*x*, *x*) = 1.0.

As the spyndex Python library works with any type of object that is compatible with overloaded operators, primitive types can be used to compute one or multiple spectral indices in a simple way. This is also true for more complex structures such as numpy.ndarray or pandas.Series objects (e.g. Figure 3). Nevertheless, real world applications usually require complex multidimensional objects that store several amounts of remote sensing data. Figure 4 shows an example where a Sentinel-2 L2A data cube for the year 2018 is retrieved as a xarray.DataArray given a specific pair of coordinates in the Hainich National Park, Germany, followed by the computation of NDVI, NIRv, and kNDVI.

### Parallel computing

Remote sensing datasets are usually large, and calculating spectral indices can be computationally expensive. For example, a single Sentinel-2 L2A image may have a data size of around 800 MB, which can increase by resampling the 20 and 60 m bands to 10 m, the highest spatial resolution of the MSI sensor. A multitemporal study using just Sentinel-2 imagery can easily overpass 1 TB of data, and appropriate scalability is required to avoid delayed processing. Computing spectral indices by using GEE objects can leverage this to scale the satellite imagery processing, which is a transparent process for users. In the specific case of the GEE JavaScript API, users may use spectral^174^, a module that extends the ASI catalogue to the Code Editor. However, when using Python, users may prefer to use arrays and data frames to have greater processing flexibility for remote sensing data. This is also useful when Machine Learning (ML) or Deep Learning (DL) modelling techniques are going to be applied.

**Fig. 5:**
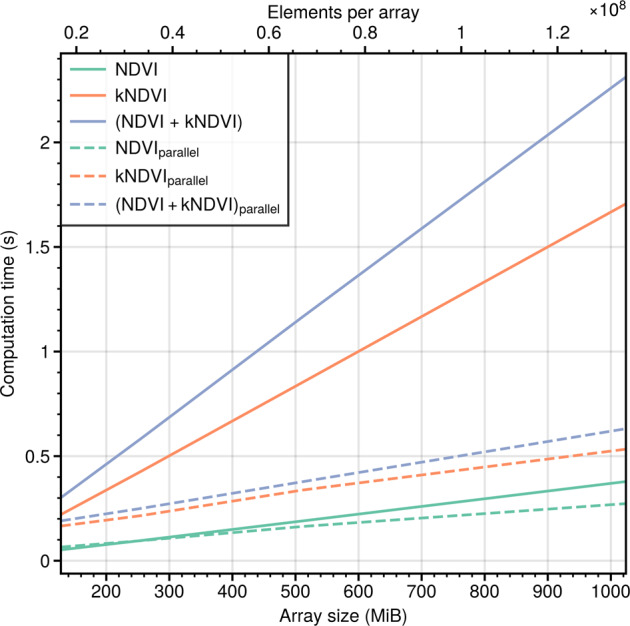
Computation time for NDVI, kNDVI and both indices at the same time (NDVI + kNDVI) for different array sizes using randomly generated array objects with chunks of 64 MiB. Performance was evaluated for serial (bold lines) and parallel (dashed lines) computing.

To leverage this scalability, dask objects can be used in combination with numpy, xarray and pandas. dask is a library for parallel computing in Python that extends the arrays and data frame objects by using chunks of data in combination with a dynamic task scheduling. Since it extends the already known arrays and data frames, dask classes are also compatible with spyndex (Table 1), allowing their calculation in parallel computing instead of the usual serial computing. By using dask, spectral indices computation can be done in parallel and multiple indices computation at once becomes an advantage over serial computing of spectral indices (Fig. 5).

### Computing spectral indices for multidimensional arrays

As Earth System Data Cubes (ESDC) are gaining strength in Earth system research^2^, spectral indices cubes can be added as new derived variables in them. Taking the vegetation application domain as example, here we present how ASI can be used for investigating the spatiotemporal spectral response of vegetation by using spectral indices at the ecosystem scale in the Hainich National Park, a Broadleaf Deciduous Forest (DBF) in central Germany^175^. The spyndex Python library was used to compute several spectral indices for Sentinel-2 imagery. To exploit the potential of data cubes for Earth system research, we will describe this example by extending the concept of ESDC to Earth System Mini Cubes (ESMC), i.e., data cubes with a small spatial extent, but with a higher spatiotemporal resolution than global data cubes).

Sentinel-2 L2A MSI data from the Hainich National Park was retrieved from the Microsoft’s Planetary Computer platform. All images were centered at the DE-Hai Eddy Covariance (EC) Tower^176^ from the Integrated Carbon Observation System (ICOS) and stored as a ESMC ${{\mathscr{C}}}_{{\rm{S}}2}(L)$ in zarr format, where *L* is the set of dimensions of ${{\mathscr{C}}}_{{\rm{S}}2}$, and *L* = {time, band, x, y}. The ESMC was preprocessed as follows: (1) the cube was cropped to a spatial extent of a 2.56 km radius around the EC tower, (2) the surface reflectance was adjusted to nadir BRDF reflectance using the *c*-factor approach^177–179^, (3) all bands were resampled to 10 m using the nearest neighbour algorithm, (4) clouds and cloud shadows were masked using the KappaMask L2A model^180^, and (5) snow was masked using the Fmask algorithm^181,182^. The temporal resolution of the dataset is 1-day and covers the period 2016–2021. However, since the revisit time of the Sentinel-2 constellation only allows for an image every 2-5 days, most of the time steps are missing.

The mini cube ${{\mathscr{C}}}_{{\rm{S}}2}(L)$ was used to compute multiple spectral indices such that it was mapped to a spectral indices mini cube ${{\mathscr{C}}}_{{\rm{S}}2}(R)$, where *R* are the set of output dimensions, and *R* = {time, index, x, y}. Since not all indices can be computed for Sentinel-2, a subset ${\Psi }_{{\rm{S}}2}\subset \Psi$ was selected such that ≪Sentinel-2≫ $\in {Z}_{\psi }$, where $\psi \in \Psi$. ${{\mathscr{C}}}_{{\rm{S}}2}(L)$ was loaded as a chunked xarray.DataArray object and the spectral indices ${\Psi }_{{\rm{S}}2}$ were computed in parallel using spyndex and dask. The additional optimizable parameters in spectral indices that required them were set to their default value according to the literature and the kernel indices were computed using ${K}_{{\rm{RBF}}}({\rho }_{i},{\rho }_{j})$ (Eq. 7) with

9 $$\sigma ={{\rm{median}}}_{{\rm{\{time,x,y\}}}}^{{\rm{\{x,y\}}}}\left(\frac{{\rho }_{i}+{\rho }_{j}}{2}\right)$$

where *ρ* is the spatiotemporal univariate mini cube of bands *i* and *j*. Note that for *σ* the temporal dimension was reduced by mapping the median function across each pixel of the cube^22,183^. This applies not only for the kNDVI, but also for the remaining kernel indices.

**Fig. 6:**
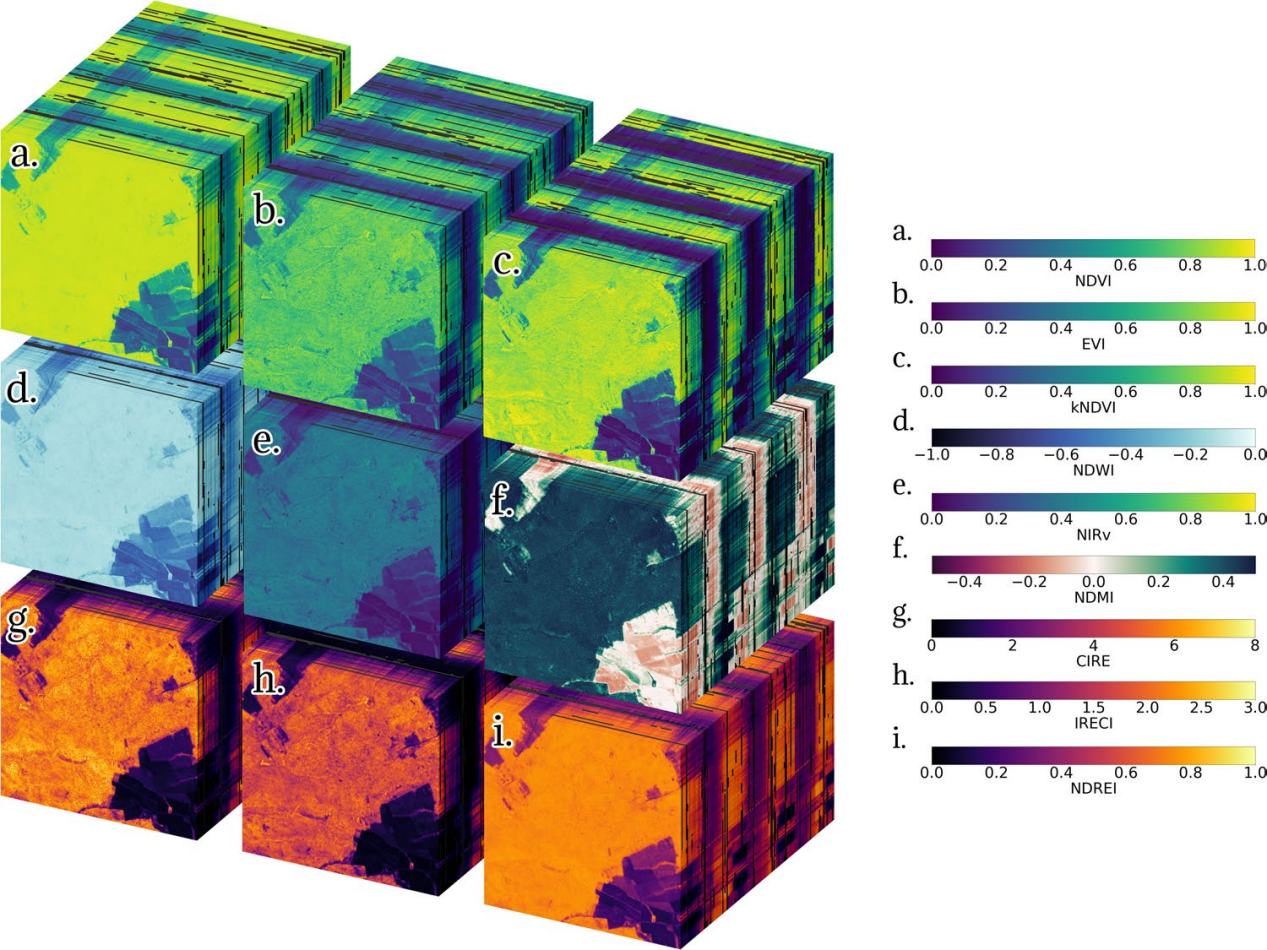
Spectral indices mini cubes computed from Sentinel-2 displaying a 2.56 km radius around the DE-Hai site. The cubes have dimensions {x = 512, y = 512, time = 195}. The initial date is 2018-07-09 (front) and the final date is 2021-09-26 (back). The displayed indices are (**a**) NDVI, (**b**) EVI, (**c**) kNDVI, (**d**) NDWI, (**e**) NIRv, (**f**) NDMI, (**g**) CIRE, (**h**) IRECI, and (**i**) NDREI. For an interactive variant of the image visit https://www.lexcube.org/.

For this document, we selected 9 spectral indices that use different bands from the visible, Red Edge, NIR and SWIR spectrum: (1) NDVI^13^, (2) EVI^90^, (3) kNDVI^22^, (4) NDWI^16^, (5) NIRv^21^, (6) Normalized Difference Moisture Index (NDMI)^148^, (7) CIRE^78^, (8) IRECI^24^, and (9) Normalized Difference Red Edge Index (NDREI)^72^. The complete set of spectral indices ψ_s2_ can be explored with the Leipzig Explorer of Earth Data Cubes (Lexcube, see https://www.lexcube.org/). Figure 6 shows the visualization of the 9 spectral indices for the DE-Hai site. Since all indices were computed from ${{\mathscr{C}}}_{{\rm{S}}2}$, the shape of the x, y, and time dimensions are maintained for each index. As the time dimension was no regular in the original cube, the timesteps are not regular in the spectral indices cubes either. Note that there are visible gaps due to the cloud-, clouds shadow- and snow-masking for all cubes (i.e., NaN values are kept as they are when indices are computed).

This example demonstrates that multiple spectral indices can be computed for high-resolution data in multiple dimensions using the Python library of ASI. While this example shows how to compute indices for Sentinel-2 data, it can be naturally extended to coarser resolution data like MODIS or to finer resolution data like satellite imagery from Planet constellations. This shows the flexibility of ASI according to the study scale. Furthermore, the potential increases with the number of indices that can be computed. Here we presented 9 spectral indices representing combinations from all spectral regions available for the Sentinel-2 platform, but hundreds more can be computed from the same bands. Additionally, indices from different application domains can be applied to each other. In this case, WIs were computed for a vegetation application domain since the water absorption bands may give meaningful insights for monitoring vegetation states.

### Spectral indices and platform capabilities

As shown before, the ASI catalogue is suitable for platforms that work on high spatial resolution datasets with a rich spectral resolution such as Sentinel-2. While this is true for the platform that was tested here, it can also serve multiple former platforms that were not mentioned, such as Landsat 1-5 with the MultiSpectral Scanner (MSS), the Advanced Spaceborne Thermal Emission and Reflection Radiometer (ASTER), MODIS, or even hyperspectral platforms like Hyperion EO-1. The use of these satellite platforms power up analyses from the global to the ecosystem scale. Nevertheless, these scales might not be sufficient according to the problem-specific objective of an Earth system study (e.g., sub-pixel sampling, recurring time-sampling, higher spectral requirements). As an example, when looking at plots with a limited spatial extent (e.g., <100 m^2^, which is the mixed area for each Sentinel-2 pixel), using airborne or drone imagery is one of the most useful approaches to take. While the spatial resolution can be significantly enhanced by using these platforms (e.g., from meters to sub-centimetres), the spectral resolution may decrease as commercial RGB cameras are used in most cases, leaving most of the spectral indices out of reach according the platform specifications. However, spectral indices in the visible spectrum can still be computed for drone missions with RGB cameras since the ASI catalogue includes several spectral indices of this kind. The subset of spectral indices in the visible spectrum can be easily retrieved as ${\Psi }_{{\rm{VIS}}}\subset \Psi$ such that ${V}_{\psi }\subseteq {V}_{{\rm{VIS}}}$, where *V*_VIS_ = {≪B≫, ≪G≫, ≪R≫}, e.g., Excess Green Index (ExG)^149^, Visible Atmospherically Resistant Index (VARI)^77^, Green Leaf Index (GLI)^106^, etc.

### Computing hyperspectral and narrow-band indices

As a matter of simplicity, the ASI catalogue currently consists of broad-band spectral indices. Some narrow-band spectral indices as well as hyperspectral indices were coerced to broad-band indices if the specific wavelength was close to a common spectral band in popular multispectral sensors (Table 2). This rules out the need of the actual reflectance in the specific wavelength, but leaves a portion of narrow-band and hyperspectral indices out of the catalogue. Some hyperspectral indices have different variants that use slightly different wavelengths in their formulas and become completely omitted. One example is the Simple Ratio (SR), which has multiple variants that can be addressed one by one when exploiting the Red Edge reflectance of vegetation^55,95,184,185^. This also applies to complete application domains, as is the case of geology indices, which generally use several SWIR narrow-bands of ASTER for lithologic mapping^186^. However, users can still use data from specific wavelengths by providing them in *P*_*ψ*_ when computing a spectral index that can be used as a hyperspectral index, such as the NDVI or the Ratio Vegetation Index (RVI). This is extensible to coerced hyperspectral indices in the catalogue, such as the Structure Insensitive Pigment Index (SIPI)^118^ or the Transformed Chlorophyll Absorption in Reflectance Index (TCARI)^83^, where users can pass the actual data from specific wavelengths as parameters for the index computation.

**Table 2:** Standard band naming and the corresponding band number for different platforms.

Band	Standard	Landsat			Sentinel		Terra/Aqua
		TM	ETM+	OLI	MSI-2A	MSI-2B	MODIS
Aerosols	A			1 (440.0)	1 (442.7)	1 (442.3)	
Blue	B	1 (485.0)	1 (485.0)	2 (480.0)	2 (492.4)	2 (492.1)	3 (469.0)
Green 1	G1						11 (531.0)
Green	G	2 (560.0)	2 (560.0)	3 (560.0)	3 (559.8)	3 (559.0)	4 (555.0)
Yellow	Y						
Red	R	3 (660.0)	3 (660.0)	4 (655.0)	4 (664.6)	4 (665.0)	1 (645.0)
Red Edge 1	RE1				5 (704.1)	5 (703.8)	
Red Edge 2	RE2				6 (740.5)	6 (739.1)	
Red Edge 3	RE3				7 (782.8)	7 (779.7)	
Near Infrared	N	4 (830.0)	4 (835.0)	5 (865.0)	8 (832.8)	8 (833.0)	2 (858.5)
Near Infrared 2	N2				8A (864.7)	8A (864.0)	
Water Vapour	WV				9 (945.1)	9 (943.2)	
Short-wave Infrared 1	S1	5 (1650.0)	5 (1650.0)	6 (1610.0)	11 (1613.7)	11 (1610.4)	6 (1640.0)
Short-wave Infrared 2	S2	7 (2215.0)	7 (2220.0)	7 (2200.0)	12 (2202.4)	12 (2185.7)	7 (2130.0)
Thermal Infrared	T	6 (11450.0)	6 (11450.0)				
Thermal Infrared 1	T1			10 (10895.0)			
Thermal Infrared 2	T2			11 (12005.0)			

Central wavelengths in nm are specified for each platform in parenthesis.

**Table 3:** Standard additional parameters naming and the spectral indices where they are used.

Parameter	Standard	Spectral Indices
Gain Factor	g	EVI
Canopy Background Adjustment	L	EVI, EVI2, MNLI, SARVI, SAVI, SAVIT
First Coefficient for the aerosol resistance term	C1	EVI, kEVI
Second Coefficient for the aerosol resistance term	C2	EVI, kEVI
*c* correction factor	cexp	OCVI
*n* exponent for the SR power operation	nexp	GDVI
*α* weighting coefficient	alpha	BWDRVI, NDPI, WDRVI
*β* calibration parameter	beta	NDSI_ns_
*γ* weighting coefficient	gamma	ARVI
*ω* weighting coefficient	omega	MBWI
*f*(Δ) adjustment factor for terrain shadows distortion	fdelta	SEVI
Soil line slope	sla	ATSAVI, SAVI2, TSAVI, WDVI
Soil line intercept	slb	ATSAVI, SAVI2, TSAVI
Photosynthetically Active Radiation	PAR	NIRvP
Soil slope parameter	k	NIRvH2
Center wavelength of band A (nm)	lambdaA	DVI+, NDGI, NIRvH2
Kernel function of A and B	kAB	kEVI, kNDVI, kRVI, kVARI

**Table 4:** Spectral indices attributes and their data types.

Attribute	Data type
short_name	string
long_name	string
application_domain	string
formula	string
bands	array (string)
platforms	array (string)
reference	string
date_of_addition	datetime
contributor	string

Data types of array items are presented in parenthesis.

### Feeding machine learning pipelines

As data driven techniques are gaining strength in Earth system research, the ASI catalogue can power up ML models. For instance, classical ML approaches are regularly used for estimating bio-physical as well as bio-chemical variables from local to global scales^183,187–189^. This includes linear regression models as well as non-linear approaches such as ensemble methods (e.g. Random Forest, Gradient Boosting, etc.), kernel methods (e.g. Support Vector Regression, SVR, Gaussian Processes, GP, etc.)^190,191^, and probabilistic methods (e.g. Naive Bayes). As Python is highly used for ML (e.g., using packages such as scikit-learn^192^; gpy, see http://sheffieldml.github.io/GPy; XGBoost, see https://xgboost.ai/; etc.), the spyndex Python library fits in the pipeline by supporting several Python objects that are often used for these methods (e.g., arrays and data frames). This means that spectral indices can be directly calculated over the dataset of interest and included as spectral features that can be even further pre-processed (e.g., computing textural features from spectral indices in 2-dimensional arrays) before being fed into a ML model. This principle applies for other pre-processing approaches in feature engineering such as dimensionality reduction. Since several spectral indices can be computed for the same input bands, a new high-dimensional feature space can be derived. As multiple indices can be highly correlated, this feature space can be reduced to a latent one, either linearly or non-linearly.

Since multiple data driven approaches also include DL techniques, ASI can also be included in DL pipelines inside Python. As spectral indices can be computed for *n*-dimensional arrays, multiple types of architectures can be used for modelling a certain variable of interest or classifying land covers. For example, a fully connected neural network can be fed with tensors that contains several spectral indices of interest for Land Use Land Change (LULC) pixel-based mapping or GPP modelling^188,189^. However, an enhanced potential of DL can be achieved by considering the spatial and temporal dimensions when we talk about remote sensing. The spatial domain is covered by using Convolutional Neural Networks (CNN) that can be fed with tensors from a 3-dimensional array. We can also grip time with Recurrent Neural Networks (RNN) architectures by adding a temporal dimension^193^. Full potential can be described by taking the spatiotemporal dimension into consideration with 4-dimensional arrays^194^. This can be effectively achieved by using different frameworks in Python, e.g., tensorflow^195^ and pytorch^196^, and exploiting the potential of ASI for handling spectral indices with *n*-dimensional data cubes.

### Using ASI outside python

The Python library is designed to manage multiple Python objects, which makes it a great tool for spectral indices computation on data frames with pandas, or data cubes by using labelled data arrays with xarray. This is extremely important for Earth system research as many variables are usually distributed along a regular spatiotemporal grid. Remote sensing data structures can then be easily transformed to fit these grids and spectral indices can be computed directly. In practice, this can be done in multiple programming languages and platforms, so their specific features as well as third-party libraries can be exploited. Given this, the ASI catalogue was already used to create a JavaScript module for the GEE Code Editor^174^, which is extensively used in Earth system research. Nevertheless, two additionally and particularly relevant programming languages to use are R and Julia.

As R is a programming language with a big geospatial user-base, multiple packages for remote sensing have been created^197^. This is the case of the sp package (see https://github.com/edzer/sp), where objects like the SpatialPixelsDataFrame can be used for storing multispectral data, permitting the computation of spectral indices. More specific packages such as raster (see https://github.com/rspatial/raster) provides users with the RasterLayer and RasterStack objects for a fully remote sensing focus. As it was mentioned before, there are already multiple R packages for computing spectral indices for these kind of objects. However, the whole potential for Earth system research can be achieved by using the ASI catalogue in combination with the stars package (see https://github.com/r-spatial/stars), specialised for spatiotemporal arrays (i.e., data cubes). On the other hand, primitive objects such as numeric types as well as vectors, arrays, and data frames can still be used for a comprehensive use of spectral indices in different objects inside R.

Julia is a programming language for high performance scientific computing, created with the goals of composability and speed of execution^198^. The latter aspect specifically makes the language suitable for large Earth system applications, and this is shown by the high number of related packages available such as EarthDataLab.jl^2^, a package developed for ESDCs, and GeoStats.jl^199^, among others. Thanks to the multiple dispatch nature of Julia, primitive objects, such as arrays, can be extended, serving as input for spatiotemporal array objects for spectral indices computation. Examples include the Zarr.jl package (see https://github.com/JuliaIO/Zarr.jl), which provides an implementation of chunked *n*-dimensional arrays, or the YAXArrays.jl package (see https://github.com/JuliaDataCubes/YAXArrays.jl), which can operate labeled data cubes from multiple data sources such as *netCDF* and *zarr* files. The thriving ML ecosystem in Julia has been growing and it now includes both production level software such as Flux.jl^200,201^ as well as state of the art libraries for scientific ML^202–204^. This allows a seamless integration of these models with specific focus on Earth system research by using spectral indices from ASI, making the exploration of different modelling techniques quick and efficient.

## Data Availability

The ASI Catalogue code is open-source and can be found at https://github.com/awesome-spectral-indices/awesome-spectral-indices and Zenodo^205^. The spyndex Python package is open-source and can be found at https://github.com/awesome-spectral-indices/spyndex. It is also available through PyPI (https://pypi.org/project/spyndex/) and conda-forge (https://anaconda.org/conda-forge/spyndex). The catalogue in CSV format can also be downloaded from the Espectro Streamlit web app, which is available at https://share.streamlit.io/davemlz/espectro/main/espectro.py. The Espectro Streamlit web app code is open-source and can be found at https://github.com/awesome-spectral-indices/espectro.
